# Hypothalamic Leptin Gene Therapy Reduces Bone Marrow Adiposity in *ob/ob* Mice Fed Regular and High-Fat Diets

**DOI:** 10.3389/fendo.2016.00110

**Published:** 2016-08-16

**Authors:** Laurence B. Lindenmaier, Kenneth A. Philbrick, Adam J. Branscum, Satya P. Kalra, Russell T. Turner, Urszula T. Iwaniec

**Affiliations:** ^1^Skeletal Biology Laboratory, School of Biological and Population Health Sciences, Oregon State University, Corvallis, OR, USA; ^2^Biostatistics Program, School of Biological and Population Health Sciences, Oregon State University, Corvallis, OR, USA; ^3^Department of Neuroscience, McKnight Brain Institute, University of Florida, Gainesville, FL, USA; ^4^Center for Healthy Aging Research, Oregon State University, Corvallis, OR, USA

**Keywords:** adipocyte, osteoblast, osteoclast, stem cell, osteoporosis, histomorphometry, rAAV

## Abstract

Low bone mass is often associated with elevated bone marrow adiposity. Since osteoblasts and adipocytes are derived from the same mesenchymal stem cell (MSC) progenitor, adipocyte formation may increase at the expense of osteoblast formation. Leptin is an adipocyte-derived hormone known to regulate energy and bone metabolism. Leptin deficiency and high-fat diet-induced obesity are associated with increased marrow adipose tissue (MAT) and reduced bone formation. Short-duration studies suggest that leptin treatment reduces MAT and increases bone formation in leptin-deficient *ob/ob* mice fed a regular diet. Here, we determined the long-duration impact of increased hypothalamic leptin on marrow adipocytes and osteoblasts in *ob/ob* mice following recombinant adeno-associated virus (rAAV) gene therapy. Eight- to 10-week-old male *ob/ob* mice were randomized into four groups: (1) untreated, (2) rAAV-Lep, (3) rAAV-green fluorescent protein (rAAV-GFP), or (4) pair-fed to rAAV-Lep. For vector administration, mice were injected intracerebroventricularly with either rAAV-leptin gene therapy (rAAV-Lep) or rAAV-GFP (9 × 10^7^ particles) and maintained for 30 weeks. In a second study, the impact of increased hypothalamic leptin levels on MAT was determined in mice fed high-fat diets; *ob/ob* mice were randomized into two groups and treated with either rAAV-Lep or rAAV-GFP. At 7 weeks post-vector administration, half the mice in each group were switched to a high-fat diet for 8 weeks. Wild-type (WT) controls included age-matched mice fed regular or high-fat diet. High-fat diet resulted in a threefold increase in MAT in WT mice, whereas MAT was increased by leptin deficiency up to 50-fold. Hypothalamic leptin gene therapy increased osteoblast perimeter and osteoclast perimeter with minor change in cancellous bone architecture. The gene therapy decreased MAT levels in *ob/ob* mice fed regular or high-fat diet to values similar to WT mice fed regular diet. These findings suggest that leptin plays an important role in regulating the differentiation of MSCs to adipocytes and osteoblasts, a process that may be dysregulated by high-fat diet. However, the results also illustrate that reducing MAT by increasing leptin levels does not necessarily result in increased bone mass.

## Introduction

Adipose tissue in bone marrow may contribute to metabolic health through its effects on local energy balance and/or its actions as an endocrine organ (1). Low bone mass is often associated with elevated bone marrow adiposity (2, 3). Bone marrow contains mesenchymal stem cells (MSCs) capable of differentiating into cells of the osteoblastic and adipocytic lineages (4). A reciprocal relationship between the number of mature osteoblasts and bone marrow adipocytes may occur as a consequence of differentiation of MSCs toward one lineage at the expense of the other lineage (5, 6). Mechanistically, peroxisome proliferator-activated receptor gamma (PPARγ), a transcription factor that plays a key role in regulation of adipogenesis and lipid uptake, has been implicated as a factor controlling MSC differentiation. Inhibition of PPARγ increases osteoblast differentiation while decreasing adipocyte differentiation. Osteoblast-targeted overexpression of PPARγ inhibits bone mass gain in male mice and increases ovariectomy-induced osteopenia in female mice (5). Furthermore, cultured adipocytes release factors capable of inducing osteoblast linage cells to differentiate into an adipocyte-like phenotype (7). Thus, excessive marrow adipose tissue (MAT) has the potential to directly and/or indirectly reduce bone mass by inhibiting osteoblast differentiation. As such, an increase in MAT may contribute to osteoporosis in conditions, such as menopause, skeletal disuse, alcohol abuse, and eating disorders (3, 8, 9).

Interestingly, anorexia and obesity both result in increased MAT (10, 11). Anorexia is commonly associated with reduced bone mineral density (BMD) and increased fracture risk. By contrast, being overweight is generally associated with increased BMD and reduced fracture risk. However, recent studies suggest that morbid obesity has negative effects on bone quality (12, 13). Thus, it is possible that the increase in MAT plays a role in the detrimental skeletal changes associated with both extremes in body weight.

Leptin, an adipokine produced in proportion to fat mass, plays a critical role in central nervous system-mediated regulation of energy homeostasis (14). Additionally, leptin plays an important positive role in skeletal growth and maturation (15, 16). Indeed, leptin signaling deficiency results in abnormal growth plate development (17) and mild osteopetrosis (18).

Leptin suppresses PPARγ expression and increases lipolysis in adipose tissue in rodents (19). In parallel, leptin increases longitudinal bone growth, osteoblastogenesis and bone formation (15, 16). Thus, the low leptin levels resulting from insufficient adipose tissue observed in anorexia may contribute to increased MAT and decreased bone formation. In contrast to anorexia, obesity typically results in increased leptin levels which, in turn, should suppress MAT. Many authors have concluded that leptin resistance plays an important role in hyperphagia and weight gain associated with obesity (20). Thus, leptin resistance may counteract the expected response to elevated leptin levels and thereby contribute to an increase in MAT during obesity. However, some new studies question whether resistance to endogenous leptin contributes to development of diet-induced obesity in mice (21, 22). If leptin resistance is a major contributor to the etiology of obesity, it may be overcome in normal rodents, at least in part, by increasing hypothalamic leptin levels. Whatever its precise role in diet-induced obesity, it is clear that leptin resistance resulting from loss of function of the leptin receptor (*db/db* mice), in addition to inducing morbid obesity, results in profound negative effects on the skeleton. Furthermore, some of these negative skeletal effects (e.g., reduced bone formation) are recapitulated, without impacting energy metabolism, following adoptive transfer of bone marrow from *db/db* mice into wild-type (WT) mice (16).

Leptin-deficient *ob/ob* and leptin receptor-deficient *db/db* mice exhibit excessive MAT in long bones (16). Short-term delivery of leptin into the hypothalamus was shown to reduce peripheral fat depots as well as MAT (23, 24). However, the long-term effects of increased leptin levels on MAT have not been well characterized. Hypothalamic leptin gene therapy has been shown to result in life-long reductions in body weight in *ob/ob* mice (25). The goal of the present study was to determine the long-duration effects of increased hypothalamic leptin, using recombinant adeno-associated virus leptin gene therapy (rAAV-Lep), on bone marrow adiposity in morbidly obese *ob/ob* mice. Given that the energy density of a diet impacts weight gain and MAT levels (26), the effects of a regular and high energy density (high fat) diet were also evaluated.

## Materials and Methods

### Experimental Animals

Eight- to 10-week-old male WT C57BL/6J (B6) and leptin-deficient *ob/ob* mice on the same genetic background were obtained from Jackson Laboratory (Bar Harbor, ME, USA). This age corresponds to peak cancellous bone volume fraction in the femur metaphysis (27). The mice were maintained in accordance with the NIH Guide for the Care and Use of Laboratory Animals and the experimental protocols were approved by the Institutional Animal Care and Use Committee at the University of Florida. The mice were housed individually in a temperature (21–23°C) and light-controlled room (lights on 6:00 a.m. to 6:00 p.m.) under specific pathogen-free conditions.

### Experiment 1: Effects of 30 Weeks of Hypothalamic Leptin Gene Therapy on Marrow Adiposity and Cancellous Bone Histomorphometry in *ob/ob* Mice

Following arrival, *ob/ob* mice were randomized by weight into four treatment groups: (1) untreated (*n* = 6), (2) control vector encoding green fluorescent protein (rAAV-GFP, *n* = 7), (3) rAAV-Lep (*n* = 8), or (4) pair-fed to rAAV-Lep (*n* = 6). The mice were maintained on standard mouse chow (LM-485, Teklad, Madison, WI, USA) and sacrificed 30 weeks following vector administration at 38–40 weeks of age. This age corresponds to a period immediately prior to a drastic increase in mortality in *ob/ob* mice – median lifespan in *ob/ob* mice is 55 weeks compared to 131 weeks in WT mice (25). The effects of treatment on hypothalamic leptin gene expression, body weight, food intake, hormone levels, organ weights, and cancellous and cortical bone architecture evaluated by microcomputed tomography in this study are detailed elsewhere (25, 28).

### Experiment 2: Effects of 15 Weeks of Hypothalamic Leptin Gene Therapy and 8 Weeks of High-Fat Diet on Marrow Adiposity and Cancellous Bone Histomorphometry in *ob/ob* Mice

Experiment 2 was conducted using WT and *ob/ob* mice. Following arrival, WT mice (*n* = 12) were maintained on regular chow (LM-485, Teklad, Madison, WI, USA; caloric density 3.4 kcal/g, 11% of kcal from fat) until 15–17 weeks of age and then randomized by weight into two groups: (1) control (*n* = 3) or (2) high-fat diet (*n* = 9). Mice in the control group continued to consume the regular diet *ad libitum* while mice in the high-fat group were placed on a high-fat diet (caloric density 4.7 kcal/g; 45% of kcal from fat, primarily from lard; Research Diets, New Brunswick, NJ, USA) fed *ad libitum*. The mice were sacrificed 8 weeks later at 23–25 weeks of age – an age corresponding to cessation of linear growth in B6 mice (27).

In conjunction, *ob/ob* mice were randomized by weight into two treatment groups: rAAV-Lep (*n* = 16) or control vector rAAV-GFP (*n* = 14). At 7 weeks post-vector administration, rAAV-GFP and rAAV-Lep mice were each divided into two groups: one group continued to consume regular diet and the other was switched to a high-fat diet as described above for WT mice. The mice were sacrificed 8 weeks later at 23–25 weeks of age (15 weeks following vector administration). The effect of the rAAV-Lep pretreatment and high-fat diet on hypothalamic leptin gene expression, body weight, food intake, organ weights, hormone levels, and cancellous and cortical bone architecture determined by microcomputed tomography are detailed elsewhere (28–30).

### Construction and Packaging of rAAV Vectors

rAAV-leptin gene therapy and rAAV-GFP vectors were constructed and packaged as previously described (31). In brief, the vector pTR-CBA-Ob *Eco*RI fragment of pCR-rOb containing rat leptin cDNA was subcloned into rAAV vector plasmid pAAVβGEnh after deleting the *Eco*RI fragment carrying the β-glucoronidase cDNA sequence. The control vector, rAAV-GFP, was similarly constructed to encode the GFP gene.

### Vector Administration

For vector administration, the mice were anesthetized with sodium pentobarbital (60 mg/kg, i.p.), placed on a Kopf stereotaxic apparatus with mouse adapter for intracerebroventricular injection, and injected intracerebroventricularly with either rAAV-Lep (9 × 10^7^ particles in 1.5 μl) or rAAV-GFP (9 × 10^7^ particles in 1.5 μl). The coordinates employed for microinjector placement in the third cerebroventricle were 0.3 mm posterior to bregma, 0.0 lateral to midline, and 4.2 mm below the dura (29).

### Tissue Collection and Analyses

At the end of each experiment mice were anesthetized with sodium pentobarbital (60 mg/kg; i.p.) and euthanized by exsanguination. Femora were excised, cleaned of soft tissue, and stored in 70% ethanol. Femora were prepared for histomorphometric evaluation as described (32). In brief, distal femora were dehydrated in graded increases of ethanol and xylene and embedded undecalcified in methyl methacrylate. Frontal sections (4 μm thick) were cut with a vertical bed microtome (Leica 2165) and affixed to slides precoated with a 1% gelatin solution. One section/animal was stained for tartrate-resistant acid phosphatase and counterstained with toluidine blue (Sigma, St Louis, MO, USA) for assessment of bone and cell-based measurements.

Histomorphometric data were collected using the OsteoMeasure System (OsteoMetrics, Inc., Atlanta, GA, USA). The sampling site for the distal femoral metaphysis was located 0.25–1.25 mm proximal to the growth plate and 0.1 mm from cortical bone. Cancellous bone measurements included bone area fraction (bone area/tissue area, %) and the derived architectural indices of trabecular number (mm^−1^), trabecular thickness (micrometer), and trabecular separation (micrometer). Measurements of MAT included overall marrow adiposity (adipose area/tissue area, %), adipocyte density (mm^−2^), and adipocyte size (micrometers^2^). Adipocytes were identified as large circular or oval-shaped cells bordered by a prominent cell membrane and lacking cytoplasmic staining due to alcohol extraction of intracellular lipids during processing. This method has been validated by fat extraction and analysis (33). Osteoblast and osteoclast perimeters were also measured and expressed as % of total bone perimeter. Osteoblasts were identified as plump cuboidal cells immediately adjacent to a thin layer of osteoid in direct contact with the bone perimeter. Osteoclasts were identified as multinucleated (two or more nuclei) cells with acid phosphatase positive (red-stained) cytoplasm in contact with the bone perimeter. Data are reported using standard two-dimensional nomenclature (34).

### Statistical Analysis

Mean responses for Experiment 1 were compared among the untreated, rAAV-GFP, rAAV-Lep, and pair-fed groups using one-way analysis of variance. For Experiment 2, the effects of treatment and diet were assessed using two-way analysis of variance. Pairwise comparisons were made using *t*-tests or the Wilcoxon–Mann–Whitney test. The required conditions for valid use of linear models were assessed using Levene’s test for homogeneity of variance, plots of residuals versus fitted values, normal quantile plots, and the Anderson–Darling test of normality. The Benjamini and Hochberg method (35) for maintaining the false discovery rate at 5% was used to adjust for multiple comparisons. Data analysis was performed using R version 3.3.2.

## Results

### Experiment 1: Effects of 30 Weeks of Hypothalamic Leptin Gene Therapy on Marrow Adiposity and Cancellous Bone Histomorphometry in *ob/ob* Mice

The effects of treatment on body weight and on cancellous bone in the distal femur metaphysis are shown in Table 1. rAAV-Lep treatment resulted in lower body weight compared to untreated, rAAV-GFP-treated, and pair-fed mice. Significant differences in cancellous bone area fraction, trabecular number, or trabecular spacing were not detected with treatment. However, trabecular thickness was lower in rAAV-Lep-treated mice compared to untreated and rAAV-GFP-treated mice, but did not differ from pair-fed *ob/ob* mice.

**Table 1:** **Effects of hypothalamic leptin gene therapy (rAAV-Lep) on body weight and cancellous bone architecture in distal femur metaphysis in *ob/ob* male mice at 30 weeks post-vector administration**.

	*ob/ob* Mice				ANOVA FDR adjusted *P*
	Untreated (*n* = 6)	rAAV-GFP (*n* = 7)	rAAV-Lep (*n* = 8)	Pair-fed (*n* = 6)	
Body weight (g)	70 ± 2	68 ± 2	29 ± 3^a^–^c^	63 ± 2	<0.001*
Bone area/tissue area (%)	8.3 ± 1.1	7.2 ± 0.9	5.3 ± 1.0	5.7 ± 0.7	0.225
Trabecular thickness (μm)	35 ± 2	34 ± 1	26 ± 2^a^^,^^b^	31 ± 2	0.009
Trabecular number (mm^−1^)	2.3 ± 0.2	2.1 ± 0.2	2.0 ± 0.2	1.8 ± 0.2	0.645
Trabecular spacing (μm)	416 ± 42	470 ± 38	542 ± 71	547 ± 73	0.588

*Data are mean ± SE*.

*Previously reported (28).

*^a^Different from untreated, P < 0.05*.

*^b^Different from rAAV-GFP, P < 0.05*.

*^c^Different from pair-fed, P < 0.05*.

**Figure 1:**
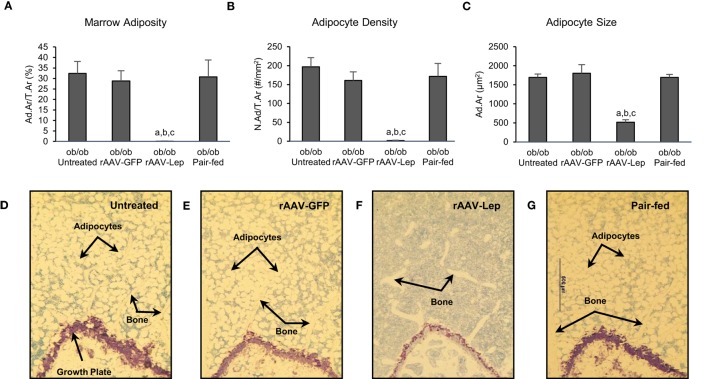
**Effects of hypothalamic leptin gene therapy (rAAV-Lep) on marrow adipose tissue in distal femur metaphysis in male *ob/ob* mice at 30 weeks post-vector administration**. Bone marrow adiposity **(A)**, adipocyte density **(B)**, and adipocyte size **(C)** were decreased with rAAV-Lep treatment. The effects of treatment on marrow adipose tissue in the femoral metaphysis can be readily appreciated in representative micrographs from an untreated **(D)**, rAAV-GFP-treated **(E)**, rAAV-Lep-treated **(F)**, and pair-fed to rAAV-Lep **(G)** mouse. Please see Figures 2C–F for representative higher magnification images. Data are mean ± SE (*n* = 6–8/group). ^a^Different from untreated, ^b^different from rAAV-GFP, and ^c^different from pair-fed, *P* < 0.05.

The effects of treatment on MAT in the distal femur metaphysis are shown in Figure 1. Marrow adiposity (adipose area/tissue area) (Figure 1A), adipocyte density (Figure 1B), and adipocyte size (Figure 1C) were lower in rAAV-Lep-treated mice compared to untreated, rAAV-GFP-treated, and pair-fed mice. Significant differences among untreated, rAAV-GFP-treated, and pair-fed mice were not detected for any of the MAT measurements. The effects of rAAV-Lep treatment on marrow adiposity can be readily appreciated in Figures 1D–G.

**Figure 2:**
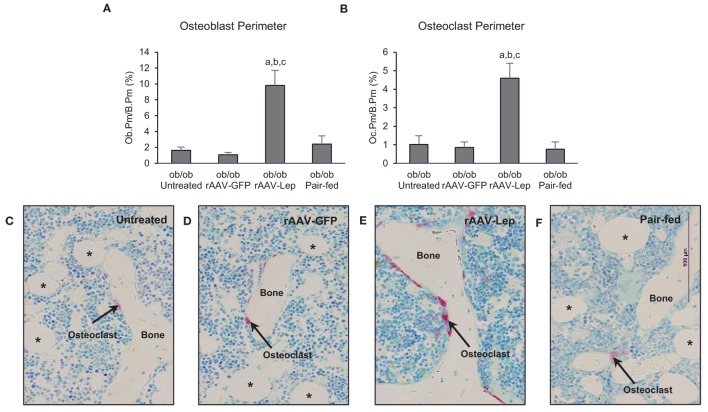
**Effects of hypothalamic leptin gene therapy (rAAV-Lep) on osteoblast perimeter, an index of bone formation, and osteoclast perimeter, an index of bone resorption, in distal femur metaphysis in male *ob/ob* mice at 30 weeks post-vector administration**. Osteoblast perimeter **(A)** and osteoclast perimeter **(B)** were increased with rAAV-Lep treatment. The effects of treatment on osteoclast perimeter, an index of bone resorption, can be readily appreciated in representative micrographs from an untreated **(C)**, rAAV-GFP-treated **(D)**, rAAV-Lep-treated **(E)**, and pair-fed to rAAV-Lep **(F)** mouse. Data are mean ± SE (*n* = 6–8/group). ^a^Different from untreated, ^b^different from rAAV-GFP, and ^c^different from pair-fed, *P* < 0.05. Asterisks demarcate adipocytes.

The effects of treatment on osteoblast perimeter and osteoclast perimeter in the distal femur metaphysis are shown in Figure 2. Osteoblast perimeter (Figure 2A) and osteoclast perimeter (Figure 2B) were higher in rAAV-Lep-treated mice compared to untreated, rAAV-GFP-treated, and pair-fed mice. Significant differences among untreated, rAAV-GFP-treated, and pair-fed mice were not detected for either of the cellular endpoints evaluated. The effects of rAAV-Lep treatment on osteoblast and osteoclast perimeter can be appreciated in Figures 2C–F.

### Experiment 2: Effects of 15 Weeks of Hypothalamic Leptin Gene Therapy and 8 Weeks of High-Fat Diet on Marrow Adiposity and Cancellous Bone Histomorphometry in *ob/ob* Mice

The effects of rAAV-Lep pretreatment and high-fat diet on body weight and on cancellous bone in distal femur metaphysis are shown in Table 2. Body weight was higher in WT mice fed high-fat diet compared to WT mice fed regular diet. Body weight was also higher in *ob/ob* mice fed high fat compared to *ob/ob* mice fed regular diet and rAAV-Lep treatment resulted in lower body weight. Cancellous bone area fraction, trabecular thickness, and trabecular number were higher and trabecular spacing was lower in WT mice fed high-fat diet compared to WT mice fed regular diet. rAAV-Lep treatment in *ob/ob* mice resulted in lower cancellous bone area fraction and trabecular thickness. Significant differences in trabecular number or trabecular spacing were not detected with treatment in the *ob/ob* mice. With the exception of trabecular thickness, which was lower, significant differences between WT mice and rAAV-Lep-treated *ob/ob* mice fed regular diets were not detected for any of the remaining cancellous endpoints evaluated.

**Table 2:** **Effects of high-fat diet and rAAV-Lep pretreatment and high fat diet on terminal body weight and cancellous bone architecture in distal femur metaphysis in male WT and *ob/ob* mice, respectively**.

				*ob/ob* Mice				
	WT mice			rAAV-GFP		rAAV-Lep		ANOVA *P*		
	Regular diet (*n* = 3)	High-fat diet (*n* = 9)	*T*-test *P*	Regular diet (*n* = 5)	High-fat diet (*n* = 9)	Regular diet (*n* = 8)	High-fat diet (*n* = 8)	Vector	Diet	Interaction
Body weight (g)	29 ± 1	38 ± 1	0.014	56 ± 2	66 ± 2	28 ± 3	33 ± 2	0.000	0.003	0.307*
Bone area/tissue area (%)	7.1 ± 1.0	11.5 ± 0.8	0.009	7.9 ± 0.9	9.9 ± 1.0	7.1 ± 0.7	6.6 ± 0.6	0.016	0.486	0.159
Trabecular thickness (μm)	30 ± 0	38 ± 1	0.009	31 ± 1	34 ± 2	25 ± 1.1^a^	25 ± 1	0.000	0.501	0.330
Trabecular number (mm^−1^)	2.3 ± 0.3	3.0 ± 0.1	0.020	2.5 ± 0.2	2.8 ± 0.1	2.8 ± 0.2	2.6 ± 0.1	0.950	0.658	0.115
Trabecular spacing (μm)	412 ± 62	297 ± 12	0.014	379 ± 29	323 ± 16	340 ± 19	363 ± 24	0.764	0.608	0.083

*Data are mean ± SE*.

*Previously reported (30).

*^a^Different from WT mice fed regular diet*.

**Figure 3:**
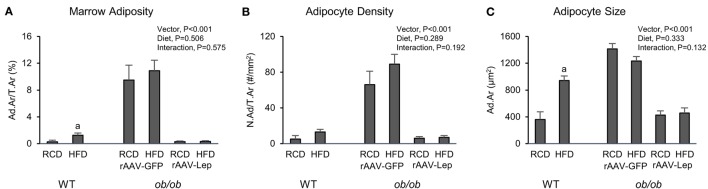
**Effects of high-fat diet and rAAV-Lep pretreatment and high-fat diet on bone marrow adiposity (A), adipocyte density (B), and adipocyte size (C) in distal femur metaphysis in male WT and *ob/ob* mice, respectively**. RCD, regular control diet. HFD, high-fat diet. Data are mean ± SE (*n* = 3–9/group). ^a^Different from RCD within WT, *P* < 0.05.

The effects of treatment on marrow adiposity in distal femur metaphysis are shown in Figure 3. Marrow adiposity and adipocyte size were higher in WT mice fed high-fat diet compared to mice fed regular diet. Significant differences in adipocyte density were not detected with diet in WT mice. rAAV-Lep treatment in *ob/ob* mice resulted in lower marrow adiposity due to lower adipocyte density as well as lower adipocyte size. Significant differences in marrow adiposity, adipocyte density, or adipocyte size were not detected between WT and rAAV-Lep-treated *ob/ob* mice fed regular diet.

## Discussion

Leptin-deficient *ob/ob* mice were heavier and had increased MAT in distal femur metaphysis compared to WT mice. Consumption of high-fat diet resulted in increased body weight in both WT mice and *ob/ob* mice but increased MAT and cancellous bone in WT mice only. rAAV-Lep treatment decreased MAT in *ob/ob* mice. The reduction in MAT in rAAV-Lep-treated *ob/ob* mice was accompanied by increases in osteoblast-lined and osteoclast-lined bone perimeter but not by an increase in cancellous bone.

Continuous and once daily intracerebroventricular administration of leptin were similarly effective in reducing MAT in long bones of *ob/ob* mice (23, 24, 36, 37). Based on lower adipocyte number and size and increased concentration of apoptosis marker caspase-3 in bone marrow adipocytes, the reduction in MAT was likely due to a combination of reduced adipocyte differentiation, increased fat oxidation, and increased adipocyte apoptosis. A similar reduction in MAT was observed following subcutaneous leptin administration (36).

In normal female rats, hypothalamic delivery of rAAV-Lep was shown to maintain lower body weight, WAT weight, and serum leptin levels (2.7 ± 0.3 versus 1.0 ± 0.1 ng/ml) for at least 18 weeks following vector administration. By contrast, rAAV-Lep transiently reduced MAT; MAT levels were reduced at 5 weeks but returned to normal levels by 10 weeks following vector administration (38). Also, hypothalamic rAAV-Lep gene therapy was ineffective in lowering MAT in ovariectomized rats (39). In the present study, MAT levels in rAAV-Lep-treated *ob/ob* mice, evaluated 15 and 30 weeks following vector administration, were much lower than age-matched *ob/ob* controls and comparable to WT mice in Experiment 2. These findings suggest that (1) while very important, the physiological actions of leptin on MAT are primarily manifested at low hormone levels and (2) hyperleptinemia has little further effect on MAT. If correct, this could help explain why some studies fail to detect a relationship between blood leptin levels and MAT (1). The findings regarding the actions of leptin on MAT are remarkably similar to the actions of the hormone on bone growth, maturation, and turnover. Whereas hypothalamic rAAV-Lep gene therapy corrected the skeletal abnormalities in *ob/ob* mice, it had minimal long-term impact on bone in rodents capable of producing leptin (16, 18, 28, 39).

It was initially hypothesized that the complex skeletal phenotype of *ob/ob* mice was due to opposing actions of peripheral and central leptin on bone formation (40, 41). However, subcutaneous and intracerebroventricular delivery of leptin were found to similarly increase bone formation in *ob/ob* mice (36). Additionally, long-duration hypothalamic leptin gene therapy was shown to normalize bone microarchitecture in *ob/ob* mice; specifically, increasing hypothalamic leptin levels resulted in increased femur length and total femur bone volume but decreased cancellous bone volume fraction in lumbar vertebra (28). These latter findings imply that, in addition to increasing longitudinal bone growth, delivery of leptin into the hypothalamus results in increased bone formation as well as increased bone resorption. Thus, an imbalance between bone formation and resorption related to local environment (e.g., precursor cell populations, mechanical loads, paracrine factors, etc.) potentially explains the contrasting phenotypes that have been identified in bones of the limb and spine in *ob/ob* mice (41).

Bone- and bone compartment-specific changes in microarchitecture in response to hormonal regulators of bone metabolism and mechanical loading environment are not unique to leptin. For example, by regulating longitudinal and radial bone growth and bone turnover balance, estrogens, and androgens contribute to sexual dimorphism of the skeleton. In this regard, administration of estrogen to growing ovariectomized rats results in shorter bones with lower total bone mass but higher site-specific cancellous bone volume (42). Ovariectomized rats also experience increased MAT expansion (39). *ob/ob* mice of both genders are hypogonadal due to reduced GnRH secretion (43), a defect that is reversed following leptin treatment (25, 29). Thus, it is possible that hypogonadism contributes to MAT expansion in *ob/ob* mice. Expansion of MAT during caloric restriction in WT mice was associated with increased circulation of glucocorticoids, while caloric restriction resulted in a further increase in the already high levels of MAT in leptin-deficient *ob/ob* mice (1, 18). These findings provide evidence that multiple factors, including leptin, regulate MAT levels.

As previously mentioned, short-duration delivery of leptin into the hypothalamus increased bone formation (36). Similarly, hypothalamic leptin gene therapy increased serum osteocalcin levels and osteoblast perimeter in lumbar vertebra of *ob/ob* mice (16, 44). In the present study, hypothalamic leptin gene therapy increased osteoblast perimeter in distal femur metaphysis in *ob/ob* mice 30 weeks following vector administration. These findings indicate that leptin promotes higher levels of bone formation prior to and following restoration of normal body weight and bone mass in *ob/ob* mice (28).

The increased cancellous bone volume fraction observed at selected skeletal sites (lumbar vertebrae) in *ob/ob* mice was initially attributed to increased bone formation, suggesting that leptin was antiosteogenic (45). However, subsequent studies consistently reported decreased bone formation in *ob/ob* mice and leptin receptor-deficient *db/db* mice, and increased bone formation following intracerebroventricular delivery of leptin, leptin gene therapy, or subcutaneous administration of leptin in *ob/ob* mice (16, 37, 44). Leptin signaling-deficient (*ob/ob* and *db/db*) mice have normal or increased osteoclast number but exhibit evidence for impaired osteoclast function (16, 18). As a consequence, these mice exhibit impaired skeletal maturation due to defective resorption of calcified cartilage. Specifically, the high cancellous bone volume fraction represents mild osteopetrosis. In the present study in *ob/ob* mice, rAAV-Lep resulted in increased osteoclast-lined bone perimeter. Thus, the failure to detect an increase in cancellous bone volume fraction in the femur metaphysis in response to higher leptin levels is likely due to parallel increases in bone formation and bone resorption.

High MAT levels in *ob/ob* mice are associated with low cancellous bone turnover (16). In the present study, rAAV-Lep resulted in increases in osteoclast-lined perimeter as well as osteoblast-lined bone perimeter and greatly reduced MAT with minimal change in cancellous bone area fraction. High MAT levels are not unique to leptin deficiency. Growth hormone deficiency in rats and mice induced by hypophysectomy or deletion of the gene for growth hormone, respectively, is also associated with high MAT and low bone turnover. In the case of growth hormone deficiency, parathyroid hormone was found to increase bone formation in hypophysectomized rats without impacting MAT levels, demonstrating that bone formation induced by bone anabolic agents is not suppressed by high levels of MAT (33). Similarly, although bone formation was increased, the absence of MAT in *kit^W/W-v^* mice did not protect against ovariectomy-induced bone loss (46). Taken together, these findings suggest that bone resorption as well as bone formation can be impacted during changes in MAT levels and interventions that target MAT may not necessarily change bone turnover balance.

A positive association between body weight and bone mass was observed in *ob/ob* as well as WT mice (30, 47). However, leptin appears to sensitize the skeleton to bone mechanical loading. This may explain why leptin-deficient mice have a low total bone mass even though they are morbidly obese and why the massive weight loss in *ob/ob* mice following leptin treatment is actually associated with a net increase in bone mass (28).

Conditional knockout of the leptin receptor in bone marrow stromal cells has been reported to result in local increases in osteogenesis and decreased adipogenesis (48). It is difficult to reconcile these findings with the skeletal phenotype of leptin receptor-deficient mice (16, 49) or with the present results demonstrating that increasing hypothalamic leptin levels profoundly reduces MAT levels. Evidence that factors secondary to leptin deficiency are responsible for the discrepancy, as suggested by Yue et al., are presently lacking and correction of many of the metabolic abnormalities associated with leptin deficiency (e.g., hyperglycemia), rather than improving, actually worsens the skeletal phenotype of *ob/ob* mice (18). Also inexplicable by Yue et al. is the finding that adoptive transfer of leptin receptor-deficient *db/db* bone marrow cells into WT mice recapitulates the low bone formation skeletal phenotype of *db/db* mice without impacting food intake or weight gain (16). It is possible that the role of leptin receptors in regulating bone metabolism depends upon stage of stromal cell differentiation (50), but this requires additional research.

A limitation of the present study is that MAT measurements were performed at a single skeletal site and MAT subtypes were not evaluated. The lipid composition and physiological function of MAT can vary with location and/or regulatory factors, such as growth hormone status (33, 51). A further limitation of most studies, including the present study, is that they have been performed housing mice at room temperature. Thermoneutral (temperature range where basal rate of energy production is at equilibrium with heat loss) in mice ranges from 26 to 34°C (52). Mild cold stress induced by room temperature housing results in dramatic cancellous bone loss at the femur metaphysis. Interestingly, mice housed at 32°C consumed ~40% less food (fed *ad libitum*) but did not differ from room temperature-housed mice in weight (53). In addition to higher bone mass, mice housed at 32°C had greatly reduced UCP-1 gene expression in brown fat, higher serum leptin, higher MAT levels due to increased adipocyte number, higher bone formation rate to due higher osteoblast perimeter, and lower osteoclast perimeter. These findings suggest that non-shivering thermogenesis substantially influences the association between leptin, MAT, and bone cells.

In summary, hypothalamic leptin gene therapy maintained low MAT levels in *ob/ob* mice fed regular or high-fat diet. The reduction in MAT was accompanied by an increase in osteoblast-lined bone perimeter but not an increase in cancellous bone volume fraction. The increase in osteoclast-lined bone perimeter suggests that the increase in bone formation was matched by an increase in bone resorption. These findings provide further evidence that a deterministic model where reducing MAT will invariably lead to increased bone volume is not tenable. As a consequence, interventions targeted at reducing MAT may not be an effective strategy for increasing bone mass.

## Author Contributions

Study design: SK. Data acquisition: LL, KP, and RT. Data analysis: AB. Data interpretation: LL, KP, AB, SK, RT, and UI. Drafting of manuscript: LL, RT, and UI. Revising manuscript content: LL, KP, AB, SK, RT, and UI. Approving final version of manuscript: LL, KP, AB, SK, RT, and UI. All authors take responsibility for the integrity of this work.

## Conflict of Interest Statement

The authors declare that the research was conducted in the absence of any commercial or financial relationships that could be construed as a potential conflict of interest.
